# 48390 Racial and Ethnic Disparities in Pediatric Kidney Transplantation - Has KAS made a difference?

**DOI:** 10.1017/cts.2021.590

**Published:** 2021-03-30

**Authors:** Olga Charnaya, Sile Yu, Aviva Goldberg, Jacqueline Garonzik-Wang, Dorry Segev, Priya S. Verghese

**Affiliations:** 1 Johns Hopkins University, School of Medicine; 2 Johns Hopkins University; 3 University of Manitoba; 4 Northwestern University Feinberg School of Medicine

## Abstract

ABSTRACT IMPACT: Evaluate the impact that the Kidney Allocation System has had on racial and ethnic disparities in pediatric deceased donor kidney transplant recipients. OBJECTIVES/GOALS: Racial and ethnic minority pediatric transplant candidates have known disparities in access to kidney transplantation. The Kidney Allocation System (KAS), implemented in 2014, was designed in part to alleviate some of these disparities thereby making transplant more equitable. We investigated the effect of KAS on reported disparities. METHODS/STUDY POPULATION: We utilized Scientific Registry of Transplant Recipients (SRTR) data to determine differences in new waitlist registrants, deceased donor (DDKT) and living donor kidney transplants (LDKT), HLA mismatch, and allograft survival among pediatric patients of different racial and ethnic backgrounds. RESULTS/ANTICIPATED RESULTS: Black pediatric patients represented 21.3% of new waitlist registrants pre-KAS and 18.9% post-KAS. Waitlist time increased for pediatric patients of all races post-KAS with the highest increase (131 days) in Asian patients (p < 0.01). The racial distribution of DDKT pre- and post-KAS was unchanged (White 38.4% vs 38.3%, Black 24.5% vs 22.5%, Hispanic 30.6% vs 31.1%, Asian 3.7% vs 4.4%, p = 0.12). The 3-yr graft failure rate is disproportionately worse in Black children compared to other races pre- and post-KAS (White 6.8% vs 5.3%, Black 14% vs 8.7%, Hispanic 8% vs 4.5%, Asian 6.6% vs 6.7%, Other 6.5% vs 2.9%) although there is a trend towards better graft survival in the post-KAS era. Graft survival worsened in Asian children in the post-KAS era (HR 2.34,95% CI 1.05 - 5.25, p=0.038). DISCUSSION/SIGNIFICANCE OF FINDINGS: Racial and ethnic disparities in pediatric ESRD patients have not been ameliorated by KAS. Children of color have longer waitlist time and are more likely to have graft failure. Alarmingly, allograft failure rate increased in Asian patients post-KAS, which merits further evaluation.

